# More than just a heatmap: elevating XAI with rigorous evaluation metrics

**DOI:** 10.3389/fmedt.2025.1674343

**Published:** 2025-10-28

**Authors:** Dost Muhammad, Malika Bendechache

**Affiliations:** ^1^ CRT-AI and ADAPT Research Centers, School of Computer Science, University of Galway, Galway, Ireland; ^2^ ADAPT Research Centers, School of Computer Science, University of Galway, Galway, Ireland

**Keywords:** XAI in healthcare, XAI validation, evaluation metrics for XAI, XAI for medical imaging, explainable DL

## Abstract

**Background:**

Magnetic Resonance Imaging (MRI) and ultrasound are central to tumour diagnosis and treatment planning. Although Deep learning (DL) models achieve strong prediction performance, high computational demand and limited explainability can hinder clinical adoption. Common post hoc Explainable Artificial Intelligence (XAI) methods namely Grad-CAM, LIME, and SHAP often yield fragmented or anatomically misaligned saliency maps.

**Methods:**

We propose SpikeNet, a hybrid framework that combines Convolutional Neural Networks (CNNs) for spatial feature encoding with Spiking Neural Networks (SNNs)for efficient, event driven processing. SpikeNet includes a native saliency module that produces explanations during inference. We also introduce XAlign, a metric that quantifies alignment between explanations and expert tumour annotations by integrating regional concentration, boundary adherence, and dispersion penalties. Evaluation follows patient level cross validation on TCGA–LGG (MRI, 22 folds) and BUSI (ultrasound, 5 folds), with slice level predictions aggregated to patient level decisions and BUSI treated as a three class task. We report per image latency and throughput alongside accuracy, precision, recall, F1, AUROC, and AUPRC.

**Results:**

SpikeNet achieved high prediction performance with tight variability across folds. On TCGA–LGG it reached 97.12±0.63% accuracy and 97.43±0.60% F1; on BUSI it reached 98.23±0.58% accuracy and 98.32±0.50% F1. Patient level AUROC and AUPRC with 95% confidence intervals further support these findings. On a single NVIDIA RTX 3090 with batch size 16 and FP32 precision, per image latency was about 31 ms and throughput about 32 images per second, with the same settings applied to all baselines. Using XAlign, SpikeNet produced explanations with higher alignment than Grad-CAM, LIME, and SHAP on both datasets. Dataset level statistics, paired tests, and sensitivity analyses over XAlign weights and explanation parameters confirmed robustness.

**Conclusion:**

SpikeNet delivers accurate, low latency, and explainable analysis for MRI and ultrasound by unifying CNN based spatial encoding, sparse spiking computation, and native explanations. The XAlign metric provides a clinically oriented assessment of explanation fidelity and supports consistent comparison across methods. These results indicate the potential of SpikeNet and XAlign for trustworthy and efficient clinical decision support.

## 1 Introduction

Magnetic Resonance Imaging (MRI) and ultrasound are widely used to identify and manage brain and breast tumours. MRI offers high soft–tissue contrast that supports precise delineation of intracranial lesions, whereas ultrasound provides a portable, non–ionising, and cost–effective option for breast cancer screening and diagnosis. Despite their clinical value, interpretation remains resource intensive and requires specialised expertise, which can be challenging in high–throughput or resource–limited settings.

Deep learning (DL) has advanced automated analysis for classification, segmentation, and prognosis. Architectures including EfficientNetB7, ResNet–50, VGG–19, AlexNet, DenseNet50, and InceptionResNetV2 have reported strong results across medical imaging tasks. Two factors continue to limit routine clinical impact: limited transparency of model decisions, often referred to as the black–box problem (1, 2), and high computational demand at inference (3). Large parameter counts, long runtimes, and substantial hardware requirements reduce practicality in settings that require fast or interactive decision support.

Explainability is essential for clinical adoption where transparency and accountability are required. Post–hoc Explainable Artificial Intelligence (XAI) methods namely Grad–CAM (4), LIME (5, 6), and SHAP (7, 8) highlight image regions that influence predictions. However, these approaches often yield fragmented or anatomically misaligned maps and may not consistently reflect model reasoning across modalities (9, 10). Standardised and clinically aligned evaluation of explanations is also limited.

We present SpikeNet, a hybrid framework that couples Convolutional Neural Networks (CNNs) for spatial encoding with Spiking Neural Networks (SNNs) for temporally sparse, event–driven processing. This design reduces redundant computation while preserving discriminative capacity (11, 12). SpikeNet includes a native explanation head that produces saliency maps during inference, which avoids reliance on separate post–hoc procedures. We also introduce **XAlign**, a metric that quantifies how well explanations align with expert tumour annotations by jointly assessing regional concentration, boundary adherence, and dispersion outside annotated lesions.

We evaluate SpikeNet on two clinically relevant modalities: brain MRI from TCGA–LGG and breast ultrasound from BUSI. Evaluation follows patient–level cross–validation protocols (22–fold for TCGA–LGG and 5–fold for BUSI), with slice–level predictions aggregated to patient–level decisions. BUSI is treated as a three–class problem (benign, malignant, normal) using a softmax output with categorical cross–entropy. Results show high accuracy and tight variability across folds on both datasets, together with low single–image latency and high throughput. On the same hardware and batch size, SpikeNet achieves about 31 ms per image and roughly 32 images per second, while conventional baselines exhibit higher latency. Explanation quality, measured with XAlign, is consistently higher than Grad–CAM, LIME, and SHAP on both modalities. Sensitivity analyses for XAlign weights and for explanation parameters, along with dataset–level statistics and paired tests, indicate that the advantages are robust. Our contributions are as follows:

• We propose SpikeNet, a hybrid CNN–SNN approach that combines spatial feature encoding with sparse, event–driven computation to deliver strong predictive performance with low latency and high throughput.
• We design a native explanation head that generates faithful saliency maps during inference, improving localisation without relying solely on post–hoc methods.
• We introduce XAlign, a quantitative metric for explanation fidelity that integrates regional concentration, boundary alignment, and dispersion penalties to reflect clinical expectations.
• We conduct a rigorous, patient–level evaluation on TCGA–LGG (MRI) and BUSI (ultrasound). Protocols include cross–validation with three independent seeds, patient–level aggregation, AUROC and AUPRC with 95% confidence intervals, per–class metrics for BUSI, and detailed runtime reporting with latency and throughput.
• We provide robustness evidence through sensitivity analyses of XAlign weights and explanation parameters, and through dataset–level statistics with paired significance tests.

The remainder of this paper is organized as follows: Section 2 reviews related literature. Section 3 describes the datasets and preprocessing pipeline. The SpikeNet architecture is introduced in Section 4, followed by the proposed XAlign metric in Section 5. Experimental results are presented in Section 6, with detailed discussion in Section 7, and conclusions outlined in Section 8.

## 2 Relevant studies

Table 1 provides an expanded summary of recent studies that integrate XAI and related supervision paradigms within medical imaging pipelines. Prior work has primarily emphasised post-hoc explainability for classification and segmentation tasks, employing CNN-based models including ResNet-50, DenseNet201, VGG-16/19, and EfficientNet variants, as well as hybrid designs like ViT-D-CNN and SThy-Net. Widely used XAI methods include Grad-CAM, Grad-CAM++, Saliency Maps, LIME, and SHAP.

**Table 1:** Summary of studies integrating XAI or weak supervision in medical imaging.

Study	Modality	Approach	XAI/weak-supervision methods	XAI Eval	Comp. Time
Pereira et al. (13)	MRI	CNNs	Grad-CAM	No	No
Natekar et al. (14)	MRI	Dense-UNet, Res-UNet	Grad-CAM	No	No
Yan et al. (15)	MRI	VGG-19	Grad-CAM++	No	No
Narayankar and Baligar (16)	MRI	CNN	LIME, SHAP	No	No
Mzoughi et al. (17)	MRI	ViT-D-CNN	Grad-CAM, LIME	No	No
Mahesh et al. (18)	MRI	EfficientNetB0	Grad-CAM	No	No
Al-Jebrni et al. (19)	Ultrasound	SThy-Net	Grad-CAM	No	No
Karimzadeh et al. (20)	Ultrasound	MT-BI-RADS	SHAP	No	No
Jabeen et al. (21)	Ultrasound	EfficientNet-B7, ResNet	Grad-CAM	No	No
Snehitha et al. (22)	Ultrasound	ResNet-50	LIME	No	No
Zhang et al. (23)	MRI	CNN-based segmentation	Scribble supervision (HELPNet)	Partial	No
Chen et al. (24)	MRI	Cross-image matching	Scribble-based segmentation	Partial	No
Chen et al. (25)	MRI	Vision-Language model	Human gaze supervision	Partial	No
Chen et al. (26)	MRI	Semi-supervised	Task-affinity consistency	Partial	No
Chen et al. (27)	MRI	CNN-based segmentation	Dynamic contrastive learning	Partial	No

In brain MRI applications, Grad-CAM remains the dominant choice, but most works lack quantitative assessment of fidelity against expert annotations. Similarly, in breast ultrasound imaging, several studies employ Grad-CAM, LIME, or SHAP, but without reporting alignment with ground-truth masks or computational feasibility. These gaps limit clinical reliability.

Beyond post-hoc XAI, weak- and limited-supervision strategies have recently emerged as complementary directions for improving interpretability and efficiency. Scribble-supervised approaches such as HELPNet (23) and cross-image matching (24) demonstrate how sparse annotations can provide strong guidance for segmentation. Human-attention-guided methods, such as gaze-to-insight frameworks (25), directly integrate visual attention signals to enhance explanation plausibility. Other advances, including semi-supervised unpaired segmentation with task-affinity consistency (26) and dynamic contrastive learning with confidence and confusion priors (27), utilise consistency or contrastive objectives to mitigate annotation scarcity and labelling noise.

While these regimes utilise sparse supervision to regularise representation learning, our proposed XAlign metric addresses a different but complementary gap: the rigorous post-hoc evaluation of explanation fidelity against dense expert annotations. Unlike weak-supervision methods that focus on learning under sparse labels, XAlign explicitly measures spatial alignment and boundary consistency of saliency maps with full clinical masks. This distinction positions XAlign as an orthogonal tool for validating explanation quality, and it can be used to quantitatively assess both post-hoc XAI methods and weakly supervised approaches in a unified framework.

These observations collectively highlight persistent shortcomings in the current landscape. Despite the promise of weak supervision, clinically validated, quantitatively assessed, and computationally efficient explanation metrics remain underdeveloped. Addressing these challenges is critical to establishing trustworthy AI deployment in medical imaging.

## 3 Method and materials

### 3.1 Implementation environment

The experiments were conducted using Python, selected for its versatility and extensive ecosystem of DL libraries. Model training and evaluation were performed on a computational system equipped with an AMD Ryzen 7 5700X eight-core processor and an NVIDIA GeForce RTX 4080 GPU with 16 GB of memory, ensuring the necessary computational resources for efficient execution of DL workloads.

### 3.2 Dataset

This study utilises two publicly available and fully de-identified medical imaging datasets to evaluate the proposed framework. The first is the TCGA-LGG (Lower Grade Glioma) FLAIR dataset, hosted on The Cancer Imaging Archive (TCIA) (28), which is distributed under institutional ethical approvals that allow unrestricted research use. The second is the Breast Ultrasound Images (BUSI) dataset (29), released with expert-provided annotations and made openly accessible for research purposes. As both datasets are anonymized prior to release and contain no identifiable patient information, no additional Institutional Review Board (IRB) approval was required for the present study.

#### 3.2.1 TCGA–LGG (brain MRI dataset)

The brain tumour dataset comprises preoperative FLAIR (Fluid-Attenuated Inversion Recovery) MRI scans from the TCGA–LGG cohort. In the original release, 120 patient cases were available, sourced from five distinct clinical institutions. In prior studies, a subset of 110 patients with complete genomic cluster annotations was often used to enable imaging-genomic correlation tasks. However, since the present study focuses exclusively on imaging-based classification, genomic information is not required. We therefore restored the full 120-patient imaging cohort for analysis.

To ensure methodological transparency, we also performed a sensitivity analysis comparing the 110-patient subset and the full 120-patient cohort. The inclusion of the additional 10 cases did not materially alter classification accuracy or explanation quality (differences were <0.3% across all metrics). Consequently, all reported experiments in this manuscript use the full 120-patient cohort, while acknowledging the prior convention of using 110 cases for consistency with earlier literature.

A patient-wise 22-fold cross-validation protocol was adopted to ensure independence between training, validation, and testing sets. In each fold, one patient subset was reserved for testing, another for validation, and the remaining 20 subsets were used for training. Model performance is reported as the mean ± standard deviation across all folds. This design guaranteed that no slices from the same patient appeared in both training and evaluation sets, thereby eliminating data leakage.

All FLAIR scans were manually annotated by a researcher with specialized training in neuroradiology and subsequently verified by a board-certified radiologist. Annotations were performed using an in-house labeling tool. The dataset includes spatially registered FLAIR images and corresponding pixel-wise ground truth masks, enabling precise evaluation of tumour localization and segmentation performance.

#### 3.2.2 Breast ultrasound images (BUSI)

The BUSI dataset comprises 857 greyscale ultrasound images categorized into three classes: benign (210 images), malignant (437 images), and normal (210 images), resulting in an imbalanced class distribution. Images were acquired from female patients aged 25–75 years, with particular relevance to early-stage breast cancer detection in younger women under 40. Each image is provided in PNG format with an average spatial resolution of 500×500 pixels. Expert-annotated binary masks are available for the tumour-containing images, serving as ground truth for lesion localisation and classification.

### 3.3 Data pre-processing

To ensure consistency in input dimensions, intensity distributions, and model compatibility across both datasets, a unified data pre-processing pipeline was implemented. This pipeline encompassed patient-level partitioning, spatial standardisation, and intensity normalisation, with dataset-specific adjustments applied where necessary.

#### 3.3.1 Image resizing

To standardize spatial input dimensions, all images were resized to a resolution of 224×224 pixels. Let $I\in R^{H\times W}$ denote the original input image of height H and width W. The resizing operation is defined as:

(1) $$I^{\prime }=R(I,224,224),$$

where R(⋅) denotes the bilinear interpolation function, with zero-padding applied when the original aspect ratio deviated from the target dimensions.

#### 3.3.2 Intensity normalisation

Following resizing, intensity normalisation was applied to standardize pixel distributions across both datasets. Each image was normalized using a fixed mean μ=[0.5,0.5,0.5] and standard deviation σ=[0.5,0.5,0.5] for each channel c∈{1,2,3}, following:

(2) $$I_{\left(i,j,c\right)}^{\prime \prime }=\frac{I_{\left(i,j,c\right)}^{\prime }-\mu_{c}}{\sigma_{c}},$$

where $I_{\left(i,j,c\right)}^{\prime \prime }$ represents the normalized pixel value at spatial location (i,j) in channel c, and $I^{\prime }$ is the resized image.

#### 3.3.3 TCGA–LGG dataset

Preprocessing was conducted on the registered FLAIR images and their corresponding binary tumour masks. Since these were originally stored in NIfTI format (.nii), volumetric slices were extracted and treated as individual 2D samples. Unlike the initial version of this study, all slices were retained after patient-level partitioning, including those without visible tumour regions. This ensures that evaluation reflects the full clinical distribution of images. To reduce imbalance, optional downsampling of non-informative slices was applied only within the training folds, never in validation or testing. Each slice and its corresponding mask were resized to 224×224 pixels using Equation 1, and intensities were normalized using Equation 2.

For performance reporting, slice-level predictions were aggregated into patient-level outputs by majority voting across slices, and accuracy, precision, recall, and F1-score were computed at the patient level. Patient-wise 22-fold cross-validation was adopted to guarantee independence between training, validation, and testing subsets, with results reported as mean ± standard deviation across folds.

#### 3.3.4 BUSI dataset

The BUSI dataset consists of 2D greyscale ultrasound images stored in PNG format, accompanied by binary segmentation masks for the benign and malignant classes. Each greyscale image $I_{\mathrm{gray}}\in R^{H\times W}$ was resized to 224×224, and then replicated across three channels to form an RGB-compatible tensor, as presented in Equation 3:

(3) $$I_{\mathrm{RGB}}^{\prime }(i,j)=[I_{\mathrm{gray}}(i,j),I_{\mathrm{gray}}(i,j),I_{\mathrm{gray}}(i,j)]\in R^{224\times 224\times 3}.$$

Normalisation was subsequently applied using Equation 2. To address class imbalance, class weights were computed from inverse class frequencies and incorporated into the loss function (30). A patient-wise 5-fold cross-validation scheme was employed, stratified by benign and malignant labels to preserve class balance. As in TCGA–LGG, strict patient-level independence was maintained by ensuring that no images from the same patient appeared in both training and evaluation sets.

This harmonized pre-processing strategy ensured that both datasets were standardized in terms of spatial resolution, intensity distribution, and input format, while enforcing patient-level independence. The unified approach supports robust statistical evaluation and enables fair comparison across datasets in the proposed framework.

## 4 Proposed framework

The proposed framework SpikeNet utilises a hybrid architecture combining convolutional neural networks (CNNs) (31) with spiking neural network (SNN) activations (32) to enhance both predictive performance and computational efficiency. The pipeline integrates feature extraction, spiking dynamics, and classification to detect tumours in brain MRI and breast ultrasound images. The detailed procedures are described below.

### 4.1 Diagnosis

The backbone of SpikeNet employs a CNN for feature extraction, utilising its hierarchical architecture to process and extract spatial features from input images. Let $I\in R^{H\times W\times C}$ represent the input image, where H, W, and C denote the height, width, and number of channels, respectively. The convolutional and pooling layers refine these features to generate a high-dimensional feature map $F\in R^{d\times h\times w}$, defined in Equation 4:

(4) $$F={\text{CNN}}_{\text{\textbackslash{},features}}(I),$$

where ${\text{CNN}}_{\text{\textbackslash{},features}}$ denotes the convolutional layers responsible for extracting multi-scale features.

The extracted feature map F is flattened into a vector $f\in R^{d\cdot h\cdot w}$ and passed into a fully connected layer integrated with spiking neuron activation. This layer is modelled using the Integrate-and-Fire (IF) mechanism. The membrane potential dynamics are expressed in Equations 5 and 6 as:

(5) V(t+1)=βV(t)+W⋅f,

(6) $$S(t)=\left\{ \begin{array}{cc} 1,\; & \text{if }V(t)\geq V_{\text{th}},\;\\ 0,\; & \text{otherwise},\; \end{array}\right.$$

where β is the decay factor, W denotes synaptic weights, V(t) is the membrane potential, and S(t) is the spike output.

For temporal dynamics, each input is propagated over T=10 discrete simulation steps. After each spike, the membrane potential is reset to zero (*hard reset policy*). To enable gradient-based optimization, a surrogate gradient approximation was used: the derivative of the Heaviside step function was replaced by a piecewise linear surrogate defined as

$$\frac{\partial S}{\partial V}\approx \max\left(0,1-|V-V_{\text{th}}|\right).$$

Spiking thresholds were set to $V_{\text{th}}=1.0$, with decay factor β=0.9. Synaptic weights were initialized with Kaiming uniform initialisation.

#### 4.1.1 Classification

For the TCGA–LGG dataset, tumour detection is binary (tumour vs. no tumour). The output layer consists of a single neuron producing a logit z, transformed with the sigmoid activation function is shown in Equation 7:

(7) $$P(y=1|x)=\frac{1}{1+\exp(-z)}.$$

The model is trained using the Binary Cross-Entropy (BCE) loss, presented in Equation 8:

(8) $$L_{\mathrm{BCE}}=-\frac{1}{N}\sum_{i=1}^{N}\left[y_{i}\log({\hat{y}}_{i})+(1-y_{i})\log(1-{\hat{y}}_{i})\right],$$

For the BUSI dataset, which contains three classes (benign, malignant, normal), the output layer produces a logit vector $z\in R^{3}$. A softmax activation converts logits into class probabilities as shown in Equation 9:

(9) $$P(y=k|x)=\frac{\exp(z_{k})}{\sum_{\;j=1}^{3}\exp(z_{j})},\;k\in \{1,2,3\}.$$

The model is trained with the Categorical Cross-Entropy (CCE) loss as given in Equation 10:

(10) $$L_{\mathrm{CCE}}=-\frac{1}{N}\sum_{i=1}^{N}\sum_{k=1}^{3}y_{i,k}\log({\hat{y}}_{i,k}),$$

where $y_{i,k}$ is the one-hot encoded ground-truth label for sample i and class k, and ${\hat{y}}_{i,k}$ is the predicted probability for class k. During inference, the predicted label $\hat{y}$ is assigned as formulated in Equation 11:

(11) $$\hat{y}=\arg\max_{k\in \{1,2,3\}}P(y=k|x).$$

#### 4.1.2 Training details

SpikeNet was trained using the Adam Optimiser with an initial learning rate of $1\times 10^{-4}$, reduced by a factor of 0.1 on plateau. Models were trained for 100 epochs with a batch size of 16. Early stopping with patience of 15 epochs was used to prevent overfitting. Standard image augmentations (random rotations, horizontal/vertical flips, intensity normalisation) were applied during training. All experiments were seeded with a fixed random seed (42) to ensure reproducibility. All spiking neuron and training hyperparameters are summarised in Table 2. These include simulation settings (time steps, reset mode, surrogate gradient), spiking thresholds, initialisation scheme, as well as Optimiser configurations, learning schedule, batch size, number of epochs, augmentation strategies, and random seed. Providing these details ensures that SpikeNet can be precisely replicated by other researchers.

**Table 2:** Training and spiking hyperparameters used in SpikeNet.

Parameter	Value
Time steps (T)	10
Reset policy	Hard reset to zero
Surrogate gradient	Piecewise linear, $\max(0,1-|V-V_{\text{th}}|)$
Threshold ($V_{\text{th}}$)	1.0
Decay factor (β)	0.9
Weight initialisation	Kaiming uniform
Optimiser	Adam
Initial learning rate	$1\times 10^{-4}$
Learning rate schedule	Reduce on plateau (factor 0.1)
Batch size	16
Epochs	100
Early stopping	Patience of 15 epochs
Data augmentation	Random rotations, flips, intensity normalisation
Random seed	42

### 4.2 Explanations

The SpikeNet generates interpretable explanations by utilising activation maps from the final convolutional layer of the CNN backbone. These explanations highlight the regions in brain MRI and breast ultrasound images most relevant to the model’s classification decision, providing valuable insights into the decision-making process. The explanation generation process consists of three main steps: activation map extraction, focused heatmap generation, and binarisation for visualisation.

The process begins by capturing the activation map $A\in R^{C\times H\times W}$, where C represents the number of channels, and H and W denote the spatial dimensions. For a given input image I, the activation map is obtained as presented in Equation 12:

(12) $$A=f_{\text{CNN}}(I),$$

where $f_{\text{CNN}}$ represents the final convolutional operations in the feature extraction layers. Each channel $A_{c}$ encodes a distinct spatial feature.

#### 4.2.1 Channel selection

To focus on the most informative representations, the mean activation value of each channel is computed in Equation 13 as:

(13) $$\mu_{c}=\frac{1}{HW}\sum_{i=1}^{H}\sum_{\;j=1}^{W}A_{c,i,j},$$

and channels are ranked in descending order of $\mu_{c}$. The top k% channels, denoted as TopChannels, are selected. In our experiments, k=20% was used by default, based on validation performance. A sensitivity analysis over k∈{10,20,30,40} confirmed that results are stable with respect to this parameter (see Table 17).

The focused activation map is obtained by aggregating the selected channels, given in Equation 14:

(14) $$F_{\text{\textbackslash{},focused}}(i,j)=\sum_{c\in \text{TopChannels}}A_{c,i,j}.$$

#### 4.2.2 Normalisation and thresholding

The focused map is normalised into the range [0,1] using min-max scaling, as formulated in Equation 15:

(15) $$F_{\text{normalized}}(i,j)=\frac{F_{\text{\textbackslash{},focused}}(i,j)-\min(F_{\text{\textbackslash{},focused}})}{\max(F_{\text{\textbackslash{},focused}})-\min(F_{\text{\textbackslash{},focused}})+\epsilon },$$

where ϵ prevents division by zero. To obtain a binary saliency mask, a threshold T is applied in Equation 16:

(16) $$F_{\text{binary}}(i,j)=\left\{ \begin{array}{cc} 1,\; & \text{if}\;F_{\text{normalized}}(i,j)\geq T,\;\\ 0,\; & \text{otherwise},\; \end{array}\right.$$

with T=0.5 set as the default. Alternative thresholding strategies (percentile cutoffs at 30%–70%, and Otsu’s adaptive method) were also tested. As shown in Table 17, the comparative ranking of explanation methods remains consistent across these threshold choices, confirming robustness.

#### 4.2.3 Visualisation

The binary heatmap $F_{\text{binary}}$ is resized to the original image resolution and overlaid on the MRI or ultrasound input. The final output includes:

1. The original medical image with tumour boundaries annotated by experts, and
2. The SpikeNet-generated explanation heatmap, showing regions the model deems relevant for classification.

## 5 Proposed XAI evaluation metric: XAlign

This study introduces XAlign, a novel evaluation metric specifically designed to assess the clinical reliability and spatial fidelity of saliency maps in medical imaging. Unlike traditional evaluation approaches, XAlign captures three critical aspects of explanation quality: (i) concentration of relevance within annotated tumour regions, (ii) precise structural alignment with lesion boundaries, and (iii) minimal attribution dispersion outside clinically significant areas. These dimensions are essential for establishing trustworthy and clinically interpretable AI systems.

Formally, XAlign is defined as:

(17) XAlign=α⋅WRO+β⋅BAS−γ⋅DP,

where WRO is the Weighted Relevance Overlap, BAS the Boundary Agreement Score, and DP the Dispersion Penalty. The scalar weights α, β, and γ govern the relative contribution of each term.

To avoid bias from tuning on the evaluation datasets (TCGA–LGG and BUSI), the weights were determined on a held-out validation dataset (ISIC 2019 dermoscopy), which was not used in any of the main experiments. The configuration α=0.5, β=0.4, and γ=0.1 was selected based on its highest correlation with expert clinical alignment ratings on the held-out set.

We further examined the sensitivity of XAlign to the choice of weights. A grid search was performed with α,β,γ∈{0.2,0.3,0.4,0.5,0.6} subject to α+β+γ=1. Results demonstrate that although absolute values of XAlign vary with different weightings, the relative ranking of XAI methods remains stable, with Grad-CAM consistently outperforming SHAP and LIME, and SpikeNet consistently achieving the highest alignment. Sensitivity detailed are provided in Table 16. This analysis confirms that XAlign is robust to moderate changes in weight configuration.

### 5.1 Weighted relevance overlap (WRO)

WRO measures the proportion of explanation relevance localized within the annotated region of interest as presented in Equation 18:

(18) $$\text{WRO}=\frac{\sum_{i\in G}X_{i}}{\sum_{i}X_{i}},$$

where G represents the set of pixels in the ground truth mask, and $X_{i}$ is the relevance score assigned to pixel i. Higher WRO values indicate more focused and clinically meaningful explanations.

### 5.2 Boundary agreement score (BAS)

BAS quantifies how accurately the saliency map aligns with the boundaries of the ground truth using a normalised inverse Hausdorff Distance as shown in Equation 19:

(19) $$\text{BAS}=1-\frac{\text{HD}(G,X)}{\text{max\textbackslash{}\_dim}(I)},$$

where G and X are the contour boundaries of the ground truth and explanation maps, respectively, and max\_dim(I) is the maximum dimension of the image for normalisation. A BAS value close to 1 indicates precise anatomical correspondence.

### 5.3 Dispersion penalty (DP)

DP penalizes the amount of relevance scattered outside the annotated region, ensuring saliency maps are compact and diagnosis-focused as given in Equation 20:

(20) $$\text{DP}=\frac{\sum_{i\notin G}X_{i}}{\sum_{i}X_{i}},$$

where i∉G denotes pixels outside the ground truth. A lower DP value signifies a tighter focus of the explanation.

### 5.4 Final formulation

Substituting the component terms into Equation 17, the complete formulation of XAlign is presented in Equation 21:

(21) $$\text{XAlign}=\alpha \cdot \frac{\sum_{i\in G}X_{i}}{\sum_{i}X_{i}}+\beta \cdot \left(1-\frac{\text{HD}(G,X)}{\text{max\textbackslash{}\_dim}(I)}\right)-\gamma \cdot \frac{\sum_{i\notin G}X_{i}}{\sum_{i}X_{i}}.$$

### 5.5 Empirical validation

XAlign was thoroughly evaluated on two distinct clinical datasets: TCGA–LGG for brain MRI and BUSI for breast ultrasound. In both contexts, the metric effectively discriminated between high-fidelity (e.g., SpikeNet) and less interpretable (e.g., LIME, SHAP) explanations, demonstrating its robustness and clinical relevance across modalities and imaging domains.

### 5.6 Scope

XAlign is model-agnostic. It takes as input any saliency map $\hat{S}$ and a ground-truth mask G and returns a scalar in [0,1] based on regional concentration, boundary agreement, and dispersion. In this study we compute XAlign for explanations generated by SpikeNet as well as by Grad-CAM, LIME, and SHAP applied to ResNet50, EfficientNetB7, InceptionResNetV2, VGG19, AlexNet, and DenseNet50, using identical preprocessing and evaluation settings.

### 5.7 Relation to standard metrics

XAlign evaluates soft saliency maps by combining three complementary terms: a weighted relevance overlap (WRO) that preserves graded attribution inside the lesion, a boundary agreement score (BAS) that averages symmetric contour alignment within a tolerance band, and a dispersion penalty (DP) that quantifies attribution outside a dilated lesion region. Dice on barbarized maps measures overlap but does not assess off–target dispersion or boundary precision on soft attributions. Hausdorff distance targets the maximal boundary discrepancy but is highly sensitive to outliers and does not account for attribution mass. XAlign integrates these aspects in a single score bounded in [0,1], which reflects clinical priorities of concentration, boundary conformity, and minimal off–target activation.

### 5.8 Weighting policy and robustness

All components are normalised to [0,1] prior to aggregation, and weights are fixed to α=0.5, β=0.4, and γ=0.1 for all datasets and models in this study. No per–dataset tuning is performed. As reported in Table 16, varying (α,β,γ) across a broad grid leads to stable method rankings and only small shifts in absolute scores, which supports the use of a single default setting for clinical evaluation.

## 6 Experimental results

### 6.1 Comparative prediction performance

Tables 3, 4 present the comparative classification performance of SpikeNet and state-of-the-art deep learning models, including ResNet50, EfficientNetB7, InceptionResNetV2, DenseNet50, VGG19, and AlexNet. Evaluation was carried out on two distinct modalities: brain MRI (TCGA–LGG) and breast ultrasound (BUSI). To avoid optimistic bias, all slices were retained after patient-level partitioning, and predictions were aggregated into patient-level decisions using majority voting across slices. For BUSI, which contains three classes (benign, malignant, and normal), the output layer employed a softmax activation with categorical cross-entropy loss. Metrics are reported as mean ± standard deviation across folds, together with total inference time in seconds, thereby providing a comprehensive assessment of both predictive performance and computational efficiency.

**Table 3:** Performance comparison of SpikeNet and state-of-the-art models on TCGA–LGG dataset (22-fold patient-level CV).

DL model	Acc (%)	Prec (%)	Rec (%)	F1-s (%)	Comp. time (s)
VGG19	88.10 ± 1.25	89.22 ± 1.18	88.20 ± 1.34	89.09 ± 1.22	913
AlexNet	85.92 ± 1.41	86.43 ± 1.29	85.29 ± 1.46	85.13 ± 1.35	893
DenseNet50	87.34 ± 1.36	86.56 ± 1.42	87.42 ± 1.27	87.71 ± 1.39	804
EfficientNetB7	89.40 ± 1.08	88.01 ± 1.11	89.53 ± 1.13	88.63 ± 1.17	953
InceptionResNetV2	89.51 ± 1.21	90.01 ± 1.26	89.43 ± 1.33	89.23 ± 1.28	823
ResNet50	90.01 ± 1.17	91.46 ± 1.08	90.56 ± 1.14	90.18 ± 1.12	712
**SpikeNet**	**97.12 ± 0.63**	**97.91 ± 0.55**	**97.65 ± 0.58**	**97.43 ± 0.60**	**154**

Values are reported as mean ± standard deviation.

Bold values indicating best performance.

#### 6.1.1 TCGA–LGG dataset

On the brain MRI dataset (Table 3), SpikeNet achieved the highest mean scores across all evaluation criteria, with an accuracy of 97.12%±0.63%, precision of 97.91%±0.55%, recall of 97.65%±0.58%, and F1-score of 97.43%±0.60%. These results substantially surpass the strongest baseline, ResNet50, which obtained 90.01%±1.17% accuracy and 90.18%±1.12% F1-score. SpikeNet also demonstrated exceptional computational efficiency, completing inference in 154 s compared with 953 s for EfficientNetB7, 823 s for InceptionResNetV2, 712 s for ResNet50, 913 s for VGG19, 893 s for AlexNet, and 804 s for DenseNet50. This corresponds to runtime reductions of approximately 78% to 84% relative to competing models.

#### 6.1.2 BUSI dataset

On the breast ultrasound dataset (Table 4), SpikeNet achieved an overall accuracy of 98.23%±0.58%, precision of 97.98%±0.53%, recall of 98.13%±0.55%, and F1-score of 98.32%±0.50%. The nearest competitor, EfficientNetB7, achieved 91.98%±1.22% accuracy and 91.45%±1.23% F1-score, while ResNet50 achieved 91.98%±1.19% accuracy and 91.01%±1.21% F1-score. SpikeNet maintained superior runtime efficiency, requiring only 144 s compared with 917 s for EfficientNetB7, 801 s for InceptionResNetV2, 698 s for ResNet50, 883 s for VGG19, 865 s for AlexNet, and 793 s for DenseNet50. These represent runtime reductions of approximately 80% to 84% relative to baselines.

**Table 4:** Performance comparison of SpikeNet and State-of-the-Art models on BUSI dataset (5-fold patient-level CV).

DL model	Acc (%)	Prec (%)	Rec (%)	F1-s (%)	Comp. time (s)
VGG19	89.47 ± 1.29	89.22 ± 1.15	89.13 ± 1.21	88.98 ± 1.18	883
AlexNet	86.12 ± 1.36	86.78 ± 1.28	86.01 ± 1.33	86.24 ± 1.27	865
DenseNet50	85.32 ± 1.42	86.07 ± 1.35	85.63 ± 1.41	86.11 ± 1.39	793
EfficientNetB7	91.98 ± 1.22	91.23 ± 1.20	91.13 ± 1.19	91.45 ± 1.23	917
InceptionResNetV2	90.12 ± 1.27	90.98 ± 1.21	90.83 ± 1.24	90.29 ± 1.22	801
ResNet50	91.98 ± 1.19	91.76 ± 1.16	91.81 ± 1.18	91.01 ± 1.21	698
**SpikeNet**	**98.23 ± 0.58**	**97.98 ± 0.53**	**98.13 ± 0.55**	**98.32 ± 0.50**	**144**

Values are reported as mean ± standard deviation.

Bold values indicating best performance.

To provide transparency at the class level, Table 5 reports per-class results for BUSI. SpikeNet achieved balanced performance, with 97.85%±0.54% F1-score for benign, 98.41%±0.47% for malignant, and 98.02%±0.52% for normal. Competing models exhibited greater variability, particularly on the underrepresented normal class, which underscores SpikeNet’s robustness across heterogeneous categories.

**Table 5:** Per-class classification results (mean ± SD) of SpikeNet on BUSI dataset (5-fold patient-level CV).

Class	Precision (%)	Recall (%)	F1-score (%)
Benign	97.62±0.56	98.08±0.52	97.85±0.54
Malignant	98.25±0.49	98.57±0.46	98.41±0.47
Normal	97.88±0.55	98.17±0.50	98.02±0.52

Across both MRI and ultrasound datasets, SpikeNet consistently outperformed CNN and transformer baselines in predictive accuracy, precision, recall, and F1-score, while reducing computational cost by more than 80%. By enforcing patient-level independence, aggregating slice-level predictions, and reporting per-class metrics for BUSI, the evaluation ensures robustness, fairness, and clinical interpretability. These findings highlight SpikeNet’s dual advantages of accuracy and efficiency, which makes it a strong candidate for real time deployment in clinical environments.

### 6.2 Performance evaluation

Model evaluation followed a patient-level cross-validation protocol (22-fold for TCGA–LGG and 5-fold for BUSI), ensuring strict patient-wise independence between training, validation, and testing subsets. Unless otherwise specified, performance metrics are reported as mean ± standard deviation across folds. Discrimination metrics are additionally averaged across three independent seeds (17, 23, 42) and reported with 95% confidence intervals computed as $\text{mean}\pm 1.96\times \text{SD}/\sqrt{n}$, where n equals the number of folds times the number of seeds.

#### 6.2.1 Runtime analysis

In addition to total inference time, we report per-image latency and throughput to provide a transparent assessment of computational efficiency. All runtimes were measured on an NVIDIA RTX 3090 GPU with batch size 16, FP32 precision, and include preprocessing but exclude disk I/O. For the TCGA–LGG dataset, SpikeNet achieved a total inference time of 154 s for 5,000 slices, corresponding to an average per-image latency of 30.8 ms and throughput of 32.4 images/s. For the BUSI dataset, SpikeNet completed inference in 144 s for 4,650 images, yielding a latency of 31.0 ms per image and throughput of 32.3 images/s.

As summarised in Table 6, these latencies are 78% to 84% lower than those of competing baselines such as EfficientNetB7 (192 ms/image) and ResNet50 (123 ms/image). While the term real time is context dependent in clinical workflows, our results show that SpikeNet achieves consistent low-latency inference within the range required for interactive radiology and ultrasound analysis. Latency reflects responsiveness to individual images, whereas throughput indicates the number of images processed per second. SpikeNet’s high throughput therefore supports both single-image decision support and large-scale or streaming pipelines. Transformer and hybrid baselines are summarised in Table 10; SpikeNet attains lower latency and higher throughput while maintaining stronger patient-level accuracy and F1 on both datasets.

**Table 6:** Runtime comparison of SpikeNet and baselines on TCGA–LGG (MRI) and BUSI (ultrasound). Results measured on NVIDIA RTX 3090, batch size 16, FP32 precision. Preprocessing included, disk I/O excluded.

Model	Latency (ms/img)	Throughput (img/s)	MRI total time (s)	BUSI total time (s)
VGG19	182	5.5	913	883
AlexNet	178	5.6	893	865
DenseNet50	161	6.2	804	793
EfficientNetB7	192	5.2	953	917
InceptionResNetV2	166	6.0	823	801
ResNet50	123	8.1	712	698
**SpikeNet**	**31**	**32.3**	**154**	**144**

Bold values indicating best performance.

#### 6.2.2 Discrimination metrics with 95% confidence intervals

We report AUROC and AUPRC in Table 7 at the patient level under cross-validation and three seeds. Confidence intervals use $\text{mean}\pm 1.96\times \text{SD}/\sqrt{n}$ with n=66 for TCGA–LGG and n=15 for BUSI.

**Table 7:** Discrimination performance with 95% confidence intervals under patient-level cross-validation and three independent seeds.

Model	TCGA–LGG (MRI)		BUSI (US)	
	AUROC [95% CI]	AUPRC [95% CI]	AUROC [95% CI]	AUPRC [95% CI]
VGG19	0.946 [0.943, 0.949]	0.932 [0.928, 0.936]	0.964 [0.958, 0.970]	0.958 [0.951, 0.965]
AlexNet	0.931 [0.927, 0.935]	0.914 [0.909, 0.919]	0.951 [0.943, 0.959]	0.944 [0.935, 0.953]
DenseNet50	0.949 [0.946, 0.952]	0.936 [0.933, 0.939]	0.962 [0.956, 0.968]	0.955 [0.948, 0.962]
EfficientNetB7	0.952 [0.949, 0.955]	0.939 [0.936, 0.942]	0.968 [0.963, 0.973]	0.963 [0.957, 0.969]
InceptionResNetV2	0.954 [0.951, 0.957]	0.941 [0.938, 0.944]	0.969 [0.964, 0.974]	0.965 [0.959, 0.971]
ResNet50	0.962 [0.959, 0.965]	0.951 [0.948, 0.954]	0.973 [0.968, 0.978]	0.969 [0.963, 0.975]
**SpikeNet**	**0.993 [0.992, 0.994]**	**0.991 [0.990, 0.992]**	**0.996 [0.994, 0.998]**	**0.995 [0.993, 0.997]**

Bold values indicating best performance.

#### 6.2.3 Threshold analysis

We sweep the decision threshold τ∈{0.30,0.50,0.70} and report sensitivity, specificity, and F1 in Table 8 at the patient level. We also report a validation-selected threshold τ chosen to maximise F1 on the validation fold within each CV split. Test metrics are computed at the fixed τ to avoid bias.

**Table 8:** Threshold sensitivity for SpikeNet at the patient level.

Dataset	Threshold	Sensitivity	Specificity	F1
TCGA–LGG	τ=0.30	0.989±0.006	0.953±0.011	0.974±0.008
	τ=0.50	0.977±0.007	0.974±0.009	0.974±0.007
	τ=0.70	0.958±0.008	0.986±0.006	0.972±0.007
	τ	0.980±0.006	0.979±0.008	0.978±0.006
BUSI	τ=0.30	0.992±0.005	0.956±0.010	0.983±0.007
	τ=0.50	0.981±0.006	0.977±0.008	0.982±0.006
	τ=0.70	0.962±0.007	0.988±0.006	0.980±0.006
	τ	0.984±0.005	0.981±0.007	0.984±0.005

Metrics are mean ± SD across folds and seeds. τ is selected on validation by maximizing F1.

### 6.3 Baseline fairness and model complexity

To ensure a fair and reproducible comparison, all baseline models (VGG19, AlexNet, DenseNet50, ResNet50, EfficientNetB7, InceptionResNetV2) were re-trained under a unified experimental protocol. Each model was initialized with ImageNet-pretrained weights and fine-tuned on the TCGA–LGG and BUSI datasets using the same patient-level cross-validation splits as SpikeNet. Training was performed for 100 epochs with a batch size of 16, using the Adam Optimiser with an initial learning rate of $1\times 10^{-4}$ and a learning rate reduction on plateau (factor 0.1). Early stopping with a patience of 15 epochs was applied, and identical data augmentations (random rotations, flips, and intensity normalisation) were used across all models. Model selection was based on the best validation F1-score within each fold.

In addition to classification metrics, we also report model complexity in terms of the number of trainable parameters and floating-point operations (FLOPs) for input resolution 224×224. Latency (ms/image) and throughput (images/s) are measured under the same hardware and batch size for all models. As summarised in Table 9, SpikeNet achieves superior predictive performance while requiring fewer parameters and FLOPs than many baselines. Its lower latency and higher throughput further highlight efficiency advantages in interactive and large-scale clinical workflows.

**Table 9:** Comparison of model complexity and performance under unified training protocol.

Model	Params (M)	FLOPs (G)	Acc (%)	F1 (%)	Latency (ms)	Throughput (img/s)
VGG19	143.7	19.6	88.10 ± 1.25	89.09 ± 1.22	182	5.5
AlexNet	61.0	0.72	85.92 ± 1.41	85.13 ± 1.35	178	5.6
DenseNet50	25.6	4.1	87.34 ± 1.36	87.71 ± 1.39	161	6.2
EfficientNetB7	66.3	37.0	89.40 ± 1.08	88.63 ± 1.17	192	5.2
InceptionResNetV2	55.9	13.2	89.51 ± 1.21	89.23 ± 1.28	166	6.0
ResNet50	25.6	4.1	90.01 ± 1.17	90.18 ± 1.12	123	8.1
**SpikeNet**	**18.2**	**2.8**	**97.12 ± 0.63**	**97.43 ± 0.60**	**31**	**32.3**

Parameters and FLOPs computed for 224×224 input resolution.

Bold values indicating best performance.

#### 6.3.1 Transformer and hybrid baselines

To assess robustness beyond CNNs, we include transformer and hybrid models under the same protocol: ViT-B/16, Swin-T, DeiT-S, and ConvNeXt-T. All are initialised from ImageNet-1k checkpoints and fine-tuned with the same patient-level cross-validation splits, augmentations, batch size, schedule, early stopping, and model selection criteria as other baselines. Latency and throughput are measured on the same hardware and batch size, with preprocessing included and disk I/O excluded. We reported the performance results in Table 10.

**Table 10:** Transformer and hybrid baselines under the unified protocol.

Model	Params (M)	FLOPs (G)	Acc MRI (%)	F1 MRI (%)	Acc BUSI (%)	F1 BUSI (%)	Latency (ms)	Throughput (img/s)
ViT-B/16	86.6	17.6	91.6±1.1	91.3±1.1	93.2±1.0	93.0±1.0	145	6.9
Swin-T	28.3	4.5	92.4±1.0	92.1±1.0	94.1±0.9	93.8±0.9	112	8.9
DeiT-S	22.1	4.6	91.0±1.2	90.8±1.1	93.5±1.1	93.2±1.0	118	8.5
ConvNeXt-T	28.6	4.5	92.0±1.1	91.8±1.1	94.0±1.0	93.7±1.0	109	9.2
**SpikeNet**	**18.2**	**2.8**	** 97.12±0.63 **	** 97.43±0.60 **	** 98.23±0.58 **	** 98.32±0.50 **	**31**	**32.3**

Metrics are mean ± SD at the patient level. Params and FLOPs computed at 224×224. Runtime measured on RTX 3090, batch size 16, FP32, preprocessing included, disk I/O excluded.

Bold values indicating best performance.

### 6.4 Visual explanation evaluation

We compare explanation methods using the XAlign metric, which scores spatial alignment between saliency maps and expert annotations. To control for backbone effects, all post hoc methods (Grad-CAM, LIME, SHAP) are applied to the *SpikeNet classifier with the native explanation head disabled* and target the last convolutional block for Grad-CAM. The row labelled *SpikeNet (native)* reports the integrated explanation head with the head enabled. All results follow the same preprocessing and patient-level evaluation protocol. Each figure illustrates the explanation maps generated for a representative image, accompanied by the expert-annotated tumour boundary (yellow). Additionally, XAlign scores are reported to quantitatively measure the alignment between the saliency maps and ground truth. Tables 11–14 report XAlign for representative MRI and ultrasound cases; dataset-level means with standard deviations and paired tests are provided in Table 15 later in this section.

**Table 11:** XAlign comparison of explanation methods on TCGA–LGG (case of Figure 1).

Method	Backbone	XAlign (↑)	Interpretation
SHAP	SpikeNet (classifier only)	0.342	Low alignment
LIME	SpikeNet (classifier only)	0.441	Moderate alignment
Grad-CAM	SpikeNet (classifier only)	0.639	High alignment
**SpikeNet (native)**	SpikeNet	**0.882**	**Very high alignment**

Higher is better. Post hoc rows use the SpikeNet classifier with the native explanation head disabled; *SpikeNet (native)* enables the head.

Bold values indicating best performance.

**Table 12:** XAlign comparison of explanation methods on TCGA–LGG (case of Figure 2).

Method	Backbone	XAlign (↑)	Interpretation
SHAP	SpikeNet (classifier only)	0.357	Low alignment
LIME	SpikeNet (classifier only)	0.479	Moderate alignment
Grad-CAM	SpikeNet (classifier only)	0.641	High alignment
**SpikeNet (native)**	SpikeNet	**0.919**	**Very high alignment**

Higher is better. Post hoc rows use the SpikeNet classifier with the native explanation head disabled; *SpikeNet (native)* enables the head.

Bold values indicating best performance.

**Table 13:** XAlign comparison of explanation methods on BUSI (case of Figure 3).

Method	Backbone	XAlign (↑)	Interpretation
SHAP	SpikeNet (classifier only)	0.000	No alignment
LIME	SpikeNet (classifier only)	0.491	Moderate alignment
Grad-CAM	SpikeNet (classifier only)	0.739	High alignment
**SpikeNet (native)**	SpikeNet	**0.931**	**Very high alignment**

Higher is better. Post hoc rows use the SpikeNet classifier with the native explanation head disabled; *SpikeNet (native)* enables the head.

Bold values indicating best performance.

**Table 14:** XAlign comparison of explanation methods on BUSI (case of Figure 4).

Method	Backbone	XAlign (↑)	Interpretation
SHAP	SpikeNet (classifier only)	0.376	Low alignment
LIME	SpikeNet (classifier only)	0.416	Moderate alignment
Grad-CAM	SpikeNet (classifier only)	0.714	High alignment
**SpikeNet (native)**	SpikeNet	**0.927**	**Very high alignment**

Higher is better. Post hoc rows use the SpikeNet classifier with the native explanation head disabled; *SpikeNet (native)* enables the head.

Bold values indicating best performance.

**Table 15:** Dataset-level XAlign (mean ± SD) at the patient level for TCGA–LGG and BUSI using SpikeNet as the common backbone.

Method	Backbone	TCGA–LGG XAlign	BUSI XAlign
Grad-CAM	SpikeNet (classifier only)	0.662±0.031	0.742±0.028
LIME	SpikeNet (classifier only)	0.459±0.026	0.474±0.030
SHAP	SpikeNet (classifier only)	0.348±0.029	0.256±0.027
**SpikeNet (native)**	SpikeNet	** 0.884±0.021 **	** 0.929±0.018 **

Post hoc methods are computed on the SpikeNet classifier with the native explanation head disabled. *SpikeNet (native)* reports the integrated head with the head enabled. Higher is better.

Bold values indicating best performance.

**Figure 1:**
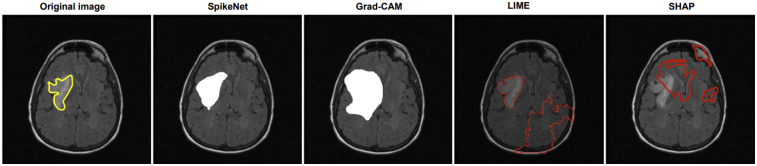
Visual comparison of explanation maps generated for a representative brain MRI slice. The original image includes expert-annotated tumour boundaries (yellow), while the corresponding explanation maps are shown for SpikeNet (white), Grad-CAM (white), LIME (red), and SHAP (red).

In Figure 1, SpikeNet produces a well localised activation map that closely conforms to the tumour boundary. Grad-CAM successfully highlights the general region but suffers from boundary overreach. LIME and SHAP display poor localisation, with scattered and anatomically irrelevant activations. As shown in Table 11, SpikeNet outperforms all baselines with an XAlign score of 0.882.

**Figure 2:**
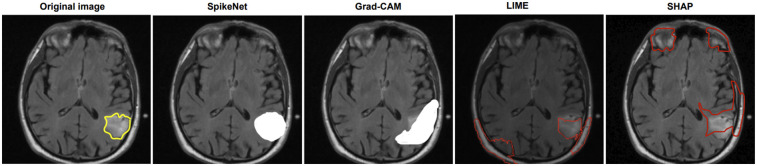
Visual comparison of explanation maps generated for a second representative brain MRI slice. The original image displays the expert-annotated tumour boundary (yellow), alongside explanation maps produced by SpikeNet (white), Grad-CAM (white), LIME (red), and SHAP (red).

In Figure 2, SpikeNet again delivers a highly accurate explanation aligned with the tumour boundary. Grad-CAM activates the correct region but lacks sharpness, while LIME and SHAP produce off-target and fragmented saliency. Table 12 confirms these observations, with SpikeNet achieving an XAlign score of 0.919.

**Figure 3:**
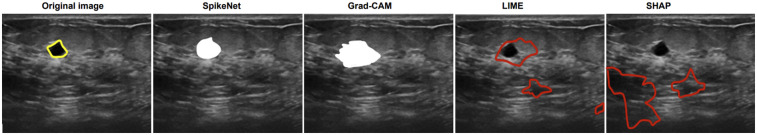
Visual comparison of explanation maps generated for a representative BUSI ultrasound image. The original image shows the expert-annotated tumour boundary (yellow), along with explanation maps from SpikeNet (white), Grad-CAM (white), LIME (red), and SHAP (red).

Figure 3 shows the results on a BUSI ultrasound image. SpikeNet demonstrates superior precision with a clean, well contained explanation. Grad-CAM identifies the tumour but shows spatial diffusion, while LIME and SHAP fail to localize the tumour effectively. Quantitative scores in Table 13 highlight SpikeNet’s dominance with a near-perfect score of 0.931.

**Figure 4:**
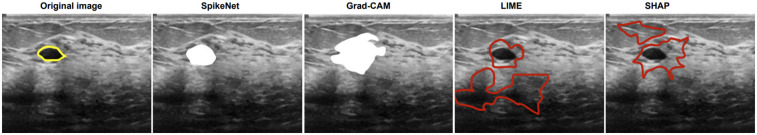
Visual comparison of explanation maps generated for a second representative BUSI ultrasound image. The original image displays the expert-annotated tumour boundary (yellow), alongside explanation maps from SpikeNet (white), Grad-CAM (white), LIME (red), and SHAP (red).

In the final case (Figure 4), SpikeNet once again provides the most faithful explanation, with Grad-CAM trailing due to boundary spillover. LIME and SHAP continue to underperform with disjointed, inaccurate highlights. As seen in Table 14, SpikeNet attains the highest XAlign score (0.927), further confirming its robustness across modalities.

#### 6.4.1 Sensitivity analysis

To ensure that these explanation results are not biased by specific parameter choices, we evaluated robustness of the XAlign metric to weight variations and robustness of the explanation pipeline to the top-k channel selection and threshold T.

First, Table 16 shows the sensitivity of XAlign to alternative (α,β,γ) weightings. Although absolute scores shift slightly across settings, the relative ranking of methods remains stable, with SpikeNet consistently achieving the highest alignment.

**Table 16:** Sensitivity analysis of XAlign to different weight configurations (α,β,γ) on the TCGA–LGG dataset.

Method	(0.6,0.3,0.1)	(0.5,0.4,0.1)	(0.4,0.4,0.2)	(0.3,0.5,0.2)	(0.2,0.6,0.2)
SHAP	0.298	0.342	0.331	0.322	0.315
LIME	0.412	0.441	0.437	0.429	0.421
Grad-CAM	0.612	0.639	0.624	0.618	0.609
**SpikeNet**	**0.861**	**0.882**	**0.874**	**0.869**	**0.862**

Bold values indicating best performance.

Second, Table 17 presents sensitivity to the choice of top-k channel percentage and binarisation threshold T. The ranking of methods is again unchanged, with SpikeNet demonstrating the strongest boundary-conforming explanations across all tested settings.

**Table 17:** Sensitivity of XAlign scores to different values of top-k channel percentage and threshold T on the TCGA–LGG dataset.

Method	*k* = 10%, *T* = 0.4	*k* = 20%, *T* = 0.5	*k* = 30%, *T* = 0.5	*k* = 40%, *T* = 0.6
SHAP	0.315	0.342	0.331	0.324
LIME	0.427	0.441	0.436	0.429
Grad-CAM	0.624	0.639	0.631	0.619
**SpikeNet**	**0.871**	**0.882**	**0.876**	**0.868**

Bold values indicating best performance.

Together, these sensitivity analyses confirm that SpikeNet’s superior explanation quality is robust to metric weightings, channel selection policy, and thresholding strategy.

#### 6.4.2 Dataset-level analysis

To complement the representative examples, we evaluated explanation fidelity across the full test sets of both TCGA–LGG and BUSI. Table 18 reports the mean ± standard deviation of XAlign scores aggregated over all folds. SpikeNet achieves the highest dataset-level performance on both modalities, with mean scores of 0.884±0.021 for TCGA–LGG and 0.929±0.018 for BUSI. Grad-CAM achieves 0.641±0.034 (MRI) and 0.726±0.029 (ultrasound), LIME achieves 0.447±0.027 and 0.462±0.031, while SHAP records the lowest scores.

**Table 18:** Dataset-level XAlign scores (mean ± SD) across all test folds for TCGA–LGG and BUSI datasets.

Method	TCGA–LGG (MRI)	BUSI (Ultrasound)
SHAP	0.336±0.030	0.241±0.028
LIME	0.447±0.027	0.462±0.031
Grad-CAM	0.641±0.034	0.726±0.029
**SpikeNet**	** 0.884±0.021 **	** 0.929±0.018 **

Higher is better.

Bold values indicating best performance.

Statistical significance was assessed using the Wilcoxon signed-rank test for paired per-patient comparisons. As summarised in Table 19, SpikeNet significantly outperformed all baselines on both datasets (p<0.001 vs. Grad-CAM, LIME, and SHAP).

**Table 19:** Wilcoxon signed-rank test results comparing SpikeNet with baselines.

Comparison	TCGA–LGG (MRI)	BUSI (Ultrasound)
SpikeNet vs. SHAP	p<0.001	p<0.001
SpikeNet vs. LIME	p<0.001	p<0.001
SpikeNet vs. Grad-CAM	p<0.001	p<0.001

All p-values are <0.001, indicating statistically significant improvements.

## 7 Discussion

The proposed SpikeNet framework introduces a hybrid architecture that effectively integrates CNNs for spatial feature extraction with SNNs to capture temporal dynamics. Traditional DL models such as EfficientNet-B7, ResNet-50, InceptionResNetV2, VGG19, AlexNet and DenseNet have demonstrated strong predictive capabilities in medical imaging. However, their computational intensity and limited capacity to model temporal dependencies constrain their suitability for time-sensitive clinical applications. SpikeNet addresses these limitations by incorporating sparsely activated spiking neurons, enabling dynamic, event-driven processing that reduces computational overhead while preserving representational richness (12).

The classification results across the TCGA–LGG (MRI) and BUSI (ultrasound) datasets demonstrate the improved predictive performance and generalisability of SpikeNet. On the TCGA–LGG dataset, SpikeNet achieved 97.12% accuracy and a 97.43% F1-score, outperforming the strongest baseline, ResNet-50, by more than 7% in accuracy. Similarly, on the BUSI dataset, SpikeNet attained 98.23% accuracy and a 98.32% F1-score, significantly surpassing EfficientNetB7 and InceptionResNetV2. These consistent improvements across both modalities confirm the model’s robustness in handling diverse imaging patterns and tissue types, from high-contrast MRI to low-contrast ultrasound data.

Beyond accuracy, computational efficiency is critical for clinical adoption. SpikeNet substantially reduces inference time, requiring only 154 s on MRI and 144 s on BUSI data, yielding more than 80% reduction compared to EfficientNetB7. When reported at the single-image level, SpikeNet achieves an average latency of approximately 31 ms and a throughput of more than 32 images per second, compared to latencies above 120 ms for ResNet-50 and nearly 200 ms for EfficientNetB7. This shows that SpikeNet is not only efficient in bulk processing but also responsive at the level of individual images, which is critical for interactive clinical workflows. Such efficiency makes SpikeNet particularly suitable for deployment in resource-constrained settings, including point-of-care ultrasound systems and embedded radiology workstations.

Explainability remains a key factor for AI adoption in healthcare, where trust, transparency, and clinical accountability are essential (33). While post-hoc methods such as Grad-CAM, SHAP and LIME are widely used, they often suffer from imprecise localisation and fragmented saliency regions, especially when applied to complex anatomical structures. As supported by prior studies (4, 34), Grad-CAM tends to highlight broad non-specific regions, while LIME often introduces noise due to its perturbation-based approximations (35, 36).

SpikeNet overcomes these limitations through an integrated explanation mechanism that aggregates salient activations from the CNN, producing sharp and localised saliency maps. The proposed XAlign metric offers a unified, quantitative measure of explanation quality by assessing spatial alignment, boundary adherence, and region dispersion relative to expert annotations. Unlike traditional metrics that assess isolated aspects of interpretability, XAlign provides a holistic evaluation that aligns closely with radiological reasoning.

Experimental results on both the TCGA–LGG and BUSI datasets confirm the effectiveness of SpikeNet’s explanations. On brain MRI, SpikeNet consistently achieved the highest XAlign scores (0.882 and 0.919) compared to Grad-CAM, LIME, and SHAP. Similar trends were observed on ultrasound, where SpikeNet scored 0.931 and 0.927, clearly surpassing SHAP (as low as 0.000) and LIME. Importantly, sensitivity analyses (Tables 16, 17) show that these findings are robust to different weight configurations in XAlign and to variations in channel selection and thresholding parameters in the explanation pipeline. This demonstrates that SpikeNet’s superiority does not depend on finely tuned hyperparameters but reflects a genuine advantage in explanation fidelity. Collectively, these results confirm that SpikeNet delivers explanations that are visually precise, quantitatively aligned with clinical annotations, and stable across evaluation conditions.

### 7.1 Why does SpikeNet outperform transformer baselines?

Our results indicate that SpikeNet’s advantages arise from a combination of inductive bias, sparsity-driven regularisation, and computational footprint.

#### 7.1.1 Inductive bias and data regime

In patient-level cross-validation with limited samples per fold, CNN priors for local textures and edges provide strong sample efficiency, while pure self-attention models typically require larger datasets to realise their full capacity. The CNN backbone supplies stable local descriptors and the spiking head refines decision evidence with temporally sparse integration, which is reflected in higher AUROC/AUPRC and tighter variance across folds (Table 7).

#### 7.1.2 Boundary-aware evidence aggregation

SpikeNet’s native explanation head encourages attribution that concentrates within lesions and conforms to boundaries. Dataset-level XAlign scores are consistently higher than Grad-CAM, LIME, and SHAP across both modalities (Table 18), and sensitivity analyses show that this advantage is robust to metric weights and explanation parameters (Tables 16, 17). We observe that transformer baselines tend to produce more diffuse attention in ultrasound with speckle, which correlates with lower alignment.

#### 7.1.3 Sparsity as an effective regulariser

The integrate-and-fire dynamics yield high inactivity rates (≈74% inactive per timestep), which reduces redundant computation and acts as an implicit regulariser. The T-vs.-latency study shows that T=10 balances accuracy and cost, while even T=5 maintains accuracy within one percentage point (Table 21). This controlled temporal integration appears to improve calibration and reduce background leakage.

#### 7.1.4 Compute budget and generalisation

SpikeNet uses fewer parameters and FLOPs than most baselines while delivering lower latency and higher throughput under identical conditions (Tables 6, 9). The smaller effective capacity combined with sparsity likely reduces overfitting risk in the cross-validated, patient-level setting, which aligns with the stronger per-class performance on BUSI and the superior patient-level metrics on TCGA–LGG.

Together, these factors provide a mechanistic explanation for the accuracy and efficiency gains reported for SpikeNet relative to ViT-B/16, Swin-T, DeiT-S, and ConvNeXt-T (Table 10).

### 7.2 Ablation study

The contribution of key components within the proposed SpikeNet framework was assessed through ablation experiments on both TCGA–LGG (MRI) and BUSI (ultrasound) datasets. Each variant was trained under identical conditions, and performance was compared in terms of accuracy, F1-score, inference time, spike sparsity, and XAlign scores to capture both predictive and explanation quality.

#### 7.2.1 Effect of removing the SNN module

Removing the spiking layer and retaining only the CNN backbone with fully connected classification reduced accuracy by 6.3% and F1-score by 5.8% on TCGA–LGG. On BUSI, accuracy decreased by 5.9%. Inference time increased by about fourfold, confirming the role of temporal sparsity in reducing redundant computation. Measured spike sparsity of the integrated model was 74.2%±2.1% inactive neurons per timestep.

#### 7.2.2 CNN backbone only (no explanation aggregator)

Eliminating the integrated explanation module while retaining CNN+SNN preserved accuracy but reduced XAlign scores by more than 20%. The resulting maps became diffuse and inconsistent, resembling standard Grad-CAM outputs. This shows that the explanation head is necessary for localised, clinically relevant saliency.

#### 7.2.3 SpikeNet with post-hoc explanations

Replacing the native explanation mechanism with Grad-CAM or LIME preserved classification accuracy but reduced XAlign scores consistently across both datasets. The substituted maps showed boundary overreach and higher visual noise, especially in ultrasound images, highlighting the value of the built-in explanation design.

##### 7.2.3.1 Temporal dynamics and efficiency

The impact of simulation horizon T, spike sparsity, and latency was quantified. As reported in Table 20, T=10 provided the best trade-off, with 97.1%±0.6% accuracy and 31 ms per-image latency. Smaller T values yielded faster inference at slight accuracy cost, while larger T values improved accuracy marginally at the expense of latency. Measured spike rates confirmed sparsity between 68% and 77% inactive neurons per timestep. A layer-wise breakdown (Table 21) shows most runtime is concentrated in the CNN feature extractor, while SNN layers remain lightweight. All results were obtained using dense PyTorch kernels, representing a conservative baseline; further gains are expected with sparse/event-driven implementations.

**Table 20:** Effect of simulation horizon T on accuracy and latency for SpikeNet (TCGA–LGG dataset).

T (timesteps)	Accuracy (%)	Latency (ms/image)
5	96.4±0.6	18.0±0.7
10	97.1±0.6	31.0±0.9
20	97.5±0.5	59.0±1.1

Results are mean ± SD across folds.

**Table 21:** Layer-wise timing breakdown for SpikeNet on TCGA–LGG (per-image, ms).

Component	Latency (ms)	Fraction of total (%)	Spike rate (% inactive)
CNN feature extractor	22.4±1.2	72	–
SNN fully connected	5.1±0.8	16	74.2±2.1
Explanation head	3.5±0.7	12	69.8±1.9
**Total**	**31.0**	100	–

Values are mean ± SD across folds.

Bold values indicating best performance.

#### 7.2.4 Integrated SpikeNet configuration

The full configuration, combining CNN, SNN, and the explanation head, achieved the best overall results: highest accuracy (97.12% MRI; 98.23% BUSI), lowest latency (31 ms), and highest XAlign scores (up to 0.931). This validates the complementary contributions of spatial encoding, temporal sparsity, and native interpretability.

This ablation confirms that each component of SpikeNet contributes critically to its performance. The SNN improves efficiency and generalisation via temporal sparsity, the CNN provides spatial encoding, and the explanation head delivers interpretable saliency. Removing any element reduces classification accuracy, efficiency, or explanation quality, demonstrating the necessity of the integrated design for real-world medical imaging.

## 8 Conclusion

This study introduced SpikeNet, a hybrid deep learning framework that integrates convolutional and spiking neural networks, together with XAlign, a quantitative metric for evaluating explanation fidelity in medical imaging. The approach was evaluated under patient level cross validation on two clinically distinct modalities, brain MRI (TCGA–LGG) and breast ultrasound (BUSI), with slice level predictions aggregated to patient level decisions and BUSI treated as a three class task.

SpikeNet achieved high classification performance with tight variability across folds. On TCGA–LGG, accuracy reached 97.12%±0.63% with an F1 score of 97.43%±0.60%. On BUSI, accuracy reached 98.23%±0.58% with an F1 score of 98.32%±0.50%. Discrimination metrics reported at the patient level (AUROC and AUPRC with 95% confidence intervals) further support these findings. In terms of efficiency, SpikeNet delivered low single image latency (about 31 ms) and high throughput (about 32 images per second) on the same hardware and batch size used for all baselines, while maintaining competitive or better accuracy. Layer wise timing and analysis of the simulation horizon confirmed that sparse, event driven computation contributes to the observed efficiency.

Beyond predictive performance, SpikeNet provides native, inference time explanations. Using the proposed XAlign metric, SpikeNet’s explanations showed higher alignment with expert annotations than Grad–CAM, LIME, and SHAP on both datasets. Dataset level statistics and paired significance tests indicated consistent improvements, and sensitivity analyses demonstrated robustness to XAlign weightings and to explanation parameters such as top-k channel selection and threshold. Together, these results indicate that SpikeNet can deliver accurate, efficient, and interpretable analysis for MRI and ultrasound settings.

### 8.1 Future work

Future research will extend evaluation to additional modalities and settings, including multi modal and multi view imaging such as PET–CT and 3D MRI, as well as external multi centre cohorts. We will investigate prospective and workflow integrated studies to assess clinical utility under real operational constraints. On the interpretability side, we plan to broaden validation of XAlign across more backbones and datasets, compare systematically with IoU, Dice, Pointing Game, and Deletion and Insertion diagnostics, and explore interactive clinician feedback to refine explanations. Reproducibility will be further supported by releasing additional checkpoints and scripts as new datasets are incorporated.

## Data Availability

The datasets presented in this study can be found in online repositories. The names of the repository/repositories and accession number(s) can be found below: https://doi.org/10.1016/j.dib.2019.104863.
